# When uncertainty becomes the norm: The Chris Hani Baragwanath Academic Hospital’s Speech Therapy and Audiology Department’s response to the COVID-19 pandemic

**DOI:** 10.4102/sajcd.v69i2.913

**Published:** 2022-08-08

**Authors:** Sadna Balton, Annika L. Vallabhjee, Stephanie C. Pillay

**Affiliations:** 1 Department of Speech Therapy and Audiology, Chris Hani Baragwanath Academic Hospital, Johannesburg, South Africa

**Keywords:** COVID-19, adaptations, autoethnography, bioecological framework, collaboration, microsystem, Speech Therapy and Audiology, South Africa, solutions, telehealth, well-being

## Abstract

**Background:**

In March 2020 the World Health Organization declared the coronavirus disease 2019 (COVID-19) a pandemic. Management of this pandemic had significant implications for clinical departments across the world. Healthcare systems were urgently required to reorganise and redesign patient care as well as repurpose staff.

**Objectives:**

We will share the lived experience of our response as speech therapy and audiology (STA) clinicians to the COVID-19 pandemic.

**Method:**

This study adopted an autoethnographic approach within Bronfenbrenner’s bioecological model to describe STA clinicians’ response to the COVID-19 pandemic.

**Results:**

Adaptations to practice were made to continue service provision whilst adhering to COVID-19 regulations. We assisted in other areas to meet the immediate needs of the hospital. Service delivery strategies consisted of a review of clinical and quality assurance protocols. We developed a telehealth service package which included a hybrid approach, within a context of digital poverty. We created resources to ensure continuity of care. Collaboration within our systems facilitated innovative solutions. Mental health and well-being of staff members were key to the response developed.

**Conclusion:**

South African healthcare systems’ inequalities were highlighted by the pandemic. The response showed that the needs of vulnerable populations were not accounted for when developing this public health response. Lessons learnt included the importance of adaptability, becoming comfortable with uncertainty and maintaining open and transparent communication. Consultation and collaboration within various levels of our healthcare system were critical in responding to the needs of patients. Commitment to compassionate leadership and staff well-being were crucial.

## Introduction

South Africa detected its first case of the coronavirus disease 2019 (COVID-19) in March 2020 (Moonasar et al., 2021), and this was the onset of uncertainty, fear, anxiety and confusion. The President of South Africa declared a National State of Disaster on 15 March 2020, followed by varied levels of lockdown as part of the measures to control and/or minimise the spread of this novel virus (Dlamini Zuma, 2020). Access to in-person healthcare and rehabilitation services was severely limited, and people avoided seeking services for fear of contracting and spreading COVID-19 (Banks, Davey, Shakespeare, & Kuper, 2020; De Biase, Cook, Skelton, Witham, & Hove, 2020; Pillay, Pienaar, Barron, & Zondi, 2021; Wilson, Crouch, Schuh, Shinn, & Bush, 2021). All outpatient services in the Speech Therapy and Audiology (STA) Department at Chris Hani Baragwanath Academic Hospital were immediately stopped and inpatient care was directed towards COVID-19 services. Outpatients who had been booked up to three months in advance had appointments cancelled. The pandemic exacerbated existing problems and concerns for people with and affected by hearing, balance, communication and swallowing disorders (Clegg, O’Flynn, & Just, 2021; Tohidas, Masuri, Bagher, & Azimi, 2020; Wilson et al., 2021). The needs of our patients required us to address the challenges that COVID-19 presented by reframing our model of care. This period of uncertainty also led to innovation, adaptation and a need for transparency and compassion amongst healthcare professionals.

## Methodology

Our response to the COVID-19 pandemic within a tertiary academic hospital’s STA department in South Africa is narrated using an autoethnographic approach (Dercon et al., 2021; Jackson & Mazzei, 2008; Marechal, 2010) within the theoretical framework of Bronfenbrenner’s bioecological model (Bronfenbrenner, 2005; Tudge, Mokrova, Hatlfield, & Karnik, 2009). Autoethnography is a reflexive consideration of the personal narrative of a group about an aspect of their experience which is embedded within a particular environment (Dercon et al., 2021; Marechal, 2010; Mendez, 2013). An autoethnographic approach, for us, facilitates a better understanding of the larger political, social and cultural phenomena experienced during the COVID-19 pandemic (Marechal, 2010; Mendez, 2013; Wall, 2008). Roy and Uekusa (2020) asserted that COVID-19 has allowed groups a unique opportunity to turn their everyday experiences, daily routines, activities and emotions into rich qualitative data in a collaborative and systemic way. Many studies published during the COVID-19 pandemic have utilised an autoethnographic approach to reflect on the lived experience of various healthcare professionals and patient groups (Das Nair et al., 2021; Hannam-Swain & Baily, 2021; Perumal et al., 2021; Wasdan, 2021; Zheng, 2021).

In fact, Roy and Uekusa (2020) argued that researchers should explore innovative, unconventional strategies to provide new insights and understanding. The authors consist of the head of the STA department and the therapists who were leading the speech therapy clinical teams during the pandemic. As current authors of this manuscript, we have deliberately chosen to write in the first person to capture and share our story as a group experience where the emphasis on self purposefully moves away from conventional and hierarchical forms of writing (Jackson & Mazzei, 2008). The decision to use the ‘I’ narrative challenges the colonial perspective which ‘creates barriers between people, places and things’ (Pillay & Kathard, 2015, p. 199). The use of ‘I’ also facilitates new ways of thinking and being (Khoza-Shangase & Mophosho, 2018). The information used to tell our story was gathered from our review of statistics, departmental reports, revised protocols and a retrospective review of personal and departmental records.

**FIGURE 1:**
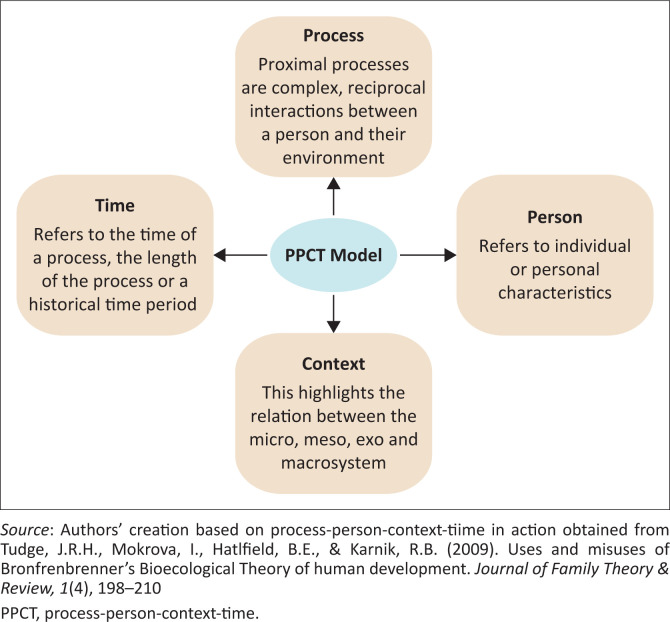
Outline of Bronfenbrenner’s process-person-context-time model.

Bronfenbrenner’s bioecological model provides a systemic, integrated approach that facilitates the understanding of the individual’s experience in a specific context and time within an interdependent system (Bronfenbrenner, 2005; Cala & Soriano, 2014; Eriksson, Ghazinour, & Hammarstrom, 2018). The COVID-19 pandemic has highlighted the interconnectedness of varied systems which has affected all domains of life (Hassan et al., 2020). It also exposed structural health inequities with increased risks to already vulnerable groups, such as the elderly and people living with disabilities (Paremoer, Nandi, Serag, & Baum, 2021; The Lancet, 2020). A systemic response to COVID-19 provided an opportunity for new knowledge to be acquired, interpreted, disseminated and applied contextually in order to build and strengthen relationships between individuals and organisations (Zięba, 2021). Figure 1 outlines Bronfenbrenner’s Process-person-context-time (PPCT) model (Bronfenbrenner, 2005; Rosa & Tudge, 2013; Tudge et al., 2009), which frames our discussion in this article.

### Person

The STA Department had a staff complement of 47 therapists and 10 auxiliary staff members during this period. The staff profile within our department consisted of white therapists, African therapists, Indian therapists and coloured therapists, with only four men in the group. More than 55% of staff members had less than five years’ experience, with 13 members having just started their community service year. Fifty-three per cent of staff members lived away from their families who resided in other provinces. Our immediate microsystem during the COVID-19 pandemic consisted of a diverse group of people who did not have extensive experience within the public healthcare sector.

### Process

The reciprocal interactions between people within the STA Department and the environment within which they function are connected through these various processes (Barach & Johnson, 2006). The STA Department is organised into five clinical teams, where each team has a designated team leader and clinic coordinators who ensure the smooth flow of services at an operational level. Inpatient and outpatient services across the lifespan are provided to patients with communication, hearing, balance and swallowing disorders. Statistics from 2019 indicated that there were a total of 35 019 contact sessions for STA. Contact sessions refer to clinical contact with patients. The Head of Department and team leaders form an executive leadership committee that engages in strategic decision-making, planning and monitoring. Our department’s functioning is guided by a strategic plan, a comprehensive annual performance plan, departmental protocols, clinical guidelines and hospital and provincial policies. Quality assurance roles, responsibilities and activities in these protocols are outlined to ensure that service standards are upheld. This includes areas such as infection control and occupational health and safety. The STA Department facilitates various activities to build a dynamic, transformative, committed and well-cared-for team with a strong sense of purpose.

### Context

Microsystems co-exist within a macrosystem (Tudge et al., 2009). The macrosystem includes legislature, government departments, social policies, economic systems, media and cultural values that influence social contexts (Newman & Newman, 2020). Our hospital is classified as a central hospital in the public healthcare sector where highly specialised, multispeciality clinical services are offered by multidisciplinary teams in an environment that fosters innovation and research (Department of Health, 2017). There are seven business units in the hospital, each under the management of a senior clinical executive who reports to the hospital’s Chief Executive Officer.

Speech Therapy and Audiology falls under Allied Health with Occupational Therapy, Physiotherapy, Dietetics, Podiatry, Social Work and Orthotics. The heads of these departments, who are not directly involved in hospital management structures, meet monthly and mainly discuss operational issues. The STA Department is interconnected with the broader healthcare system within the province. At a provincial level, we are represented on the executive committee for STA as well as the Rehabilitation Forum which has representation from Occupational Therapy, Physiotherapy and STA. These structures fall under the directorate for Oral Health and Therapeutic Services in Gauteng. The STA Department is therefore integrally linked and accountable to broader structures within the hospital and province. Furthermore, the STA Department provides a clinical platform for STA students from the University of the Witwatersrand and hosts students doing their electives or observations from other universities in the country. Our microsystem is therefore extensive, with our mesosystem consisting of varied levels of interaction and engagement. At a macrosystem, services are informed and planned in accordance with the burden of disease, sociodemographic conditions (Pillay, Tiwari, Kathard, & Chiktay, 2020) and the linguistic and cultural diversity of our patients (Khoza-Shangase & Mophosho, 2018).

### Time

Time as defined within the PPCT model will be highlighted in our discussion through identifying the activities that took place within a specific time period, March 2020 to December 2021. The continuous adaptations to processes within the microsystem were also defined by changes within the macrosystem at a particular time, e.g., adapting to the changes in legislature and its impact on services.

## Findings and discussion

The clinical microsystem assessment tool in Table 1 (Johnson, 2010) identifies the characteristics of high-performing microsystems (Barach & Johnson, 2013).

**TABLE 1:** Clinical microsystem assessment tool.

Characteristic	Description
**Leadership**	
Leadership	The role of leaders is to balance setting and reaching collective goals, and to empower individual autonomy and accountability, through building knowledge, respectful action, reviewing and reflecting.
Organisational support	The larger organisation looks for ways to support the work of the microsystem and coordinate the hand-offs between microsystems.
**Staff**	
Staff focus	There is elective hiring of the right kind of people. The orientation process is designed to fully integrate new staff into culture and work roles. Expectations of staff members are high regarding performance, continuing education, professional growth and networking.
Education and training	All clinical microsystems have responsibility for the ongoing education and training of staff and for aligning daily work roles with training competencies. Academic clinical microsystems have the additional responsibility of training students.
Interdependence	The interaction of staff members is characterised by trust, collaboration, willingness to help each other, appreciation of complementary roles, respect and recognition that all contribute individually to a shared purpose.
**Patients**	
Patient focus	The primary concern is to meet all patient needs: caring, listening, educating, responding to specific requests, innovating to meet the patients’ needs and smooth service flow.
Community focus	The microsystem is a resource for the community; the community is a resource to the microsystem; the microsystem establishes excellent and innovative relationships with the community.
**Performance**	
Performance results	Performance focuses on patient outcomes, avoidable costs, streamlining delivery, using data feedback and frank discussion about performance.
Process improvement	An atmosphere for learning and redesign is supported by the continuous monitoring of care, use of benchmarking, frequent tests of change and a staff that has been empowered to change.
**Information and information technology**	
Information and information technology	Technology facilitates effective communication and multiple formal and informal channels are used to keep everyone informed all the time, listen to everyone’s ideas and ensure that everyone is connected on important topics.

*Source*: Johnson, J. (2010). The health care interdisciplinary context: A focus on the microsystem concept. In B. Freshman, L. Rubino, &Y. Chassiakos (Eds.), *Collaboration across the disciplines* (pp. 19–41). Burlington, MA: Jones & Bartlett Publishers.

Adhering to Table 1, the discussion of our response to the COVID-19 pandemic focuses on leadership, staff and patients with performance and information integrated into these areas.

### Leadership

A crisis requires ‘positive leadership amid unprecedented change, to lead and function as active, authentic, aware, adaptive, flexible, as well as trusted, engaged and compassionate communicators’ (Hill, Butnoris, Dowling, Macolino, & Patel, 2020, p. 294). At the onset of Level 5 lockdown, we were instructed by hospital management to stop all nonessential services, which included all outpatient STA services. This sudden change in operations required us to rethink our purpose and function within the hospital. Mather (2020) recommended that leaders should go back to their core values and purpose at a time of crisis. Our vision and mission as a department, as displayed in Table 2, therefore guided our decision-making during this period.

**TABLE 2:** Vision, mission and values of the Speech Therapy and Audiology Department.

Vision	Mission	Values
To be the centre of excellence in providing services that enrich the lives of its community	To improve the quality of life for people with and affected by communication, hearing, balance, feeding and swallowing difficulties guided by the principles of best practice, research and national priorities	Patient-centred care Accountability Empowerment Transparency Efficiency Integrity

According to Heath, Sommerfield and Von Ungern-Sternberg (2020), keeping staff informed serves the purpose of building resilience through a sense of increased control over one’s environment and displays a commitment to engage and support staff directly. An urgent staff meeting was called by the Head of Department to relay information about the directive received and its impact on services and staff. Staff members communicated their fears about COVID-19 in this meeting but added that they wanted to contribute to the hospital’s response. Unfortunately, at this initial stage the hospital’s response was not clearly communicated by management. Organisational support in the hospital (Barach & Johnson, 2013) was activated only a few weeks later through the development of a steering committee under the leadership of the Head of Internal Medicine. This committee met biweekly via the Microsoft Teams platform. Statistics on the number of COVID-19 admissions, staff positivity rates, logistical challenges, personal protective equipment (PPE) and ward reallocations were some of the areas discussed. These meetings facilitated improved relations with various stakeholders within the hospital and continued to build the identity of rehabilitation. The Head of the STA Department attended these meetings and provided feedback to staff members on a regular basis. These sessions provided an opportunity for staff to be informed and to participate in decision-making. In a crisis, employees depend on organisational leaders for information, and effective communication of critical decisions (Sanders, Ngugen, Boukenogne, Rafferty, & Schwarz, 2020). Initially, all information received through various platforms was shared with staff members to reduce the anxiety associated with COVID-19. However, this influx of information became very overwhelming. We refined our communication strategy as our knowledge, skills and understanding of COVID-19 improved. We agreed on a set of principles to guide our communication in terms of the nature, frequency, time and platforms that would be used.

Mather (2020) and Bronfenbrenner (2005) stated that during a crisis or a specific historical event, there is a need to act with urgency as wasted time and delays can be damaging. During this period there was no communication received from the National and Provincial Rehabilitation Directorates about the role of rehabilitation personnel in and during COVID-19. It was therefore critical for decisions to be made at an STA departmental level to ensure that we continued functioning with a sense of purpose within the health system. We engaged with our exosystem to review material generated by international and national associations and the Health Professions Council of South Africa. This facilitated the development of standard operating procedures, adaptation of protocols and creation of resources to guide clinical practice. Because of the urgency of this response, our protocols were developed in a silo and did not include consultation with other disciplines in the hospital. Upon reflection, it was evident that to ensure a unified approach, this process should have been guided by the leadership of National and Provincial Rehabilitation structures to ensure a more comprehensive and integrated response. Adaptable, transparent and compassionate leadership (De Zulueta, 2016; Poorvakos, 2016) was key in helping us lead during a period marked with uncertainty.

### Person (staff)

Research has shown an increase in levels of stress, anxiety and depression of healthcare professionals during the COVID-19 pandemic (Feijt, De Kort, Bongers, Bierbooms, Westerink, & IJsselsteijn, 2020; Prasad et al., 2021; Zhang et al., 2020). A valued member of the microsystem is one that feels that their needs are taken into consideration by the organisation (Johnson, 2010). The STA Department therefore focused on the well-being of our staff. This was achieved through training and development, creation of a supportive and safe environment and a culture that facilitated trust, transparency, empathy, open-communication and teamwork (Barach & Johnson, 2006).

#### Training and development

Healthcare professionals should be provided with evidence-based knowledge and tools to cope with the impact of COVID-19 (Rana, Mukhtar, & Mukhtar, 2020). A systematic review by Cag et al. (2020) reported that supportive learning environments facilitate resilience and support mental health of healthcare professionals, during and after a pandemic. Training that was done in our department focused on understanding COVID-19 and its impact on speech, language, eating and drinking, balance and hearing. Infection control, occupational health and safety procedures and well-being strategies were also included. The details of our training programme are presented in Table 3.

**TABLE 3:** Speech Therapy and Audiology training and development programme.

Clinical evidence	Staff well-being	Staff safety
COVID-19 research: disease profile and clinical presentation	Coping during a pandemic	National regulations
Ethical decision-making in the public health sector during COVID-19	Toolkit for emotional coping for healthcare staff	Donning and doffing
Clinical guidelines, webinars and courses	Mindfulness	Mask usage
Telehealth	Mental health matters in the workplace: navigating the tsunami of mental health	Hand washing
A disability inclusive response to COVID-19	Managing stress	Hospital occupational health and safety training: symptom monitoring, isolation and risk assessments
Human rights and ethics: was this compromised during COVID-19?	Destigmatising mental health in the workplace	COVID vaccine roll-out

#### Staff safety

Providing a safe work environment is a matter of social justice, and healthcare professionals need to be safe to provide care for others (Zungu et al., 2021). Recommendations made by the World Health Organization (WHO) (2021) and the South African Department of Employment and Labour (2020) included the implementation of work shifts and alternating work weeks to limit interactions across coworkers as part of measures towards reducing infections (Carnevale & Hatak, 2020; Sanchez-Taltavull, Castelo-Szekely, Candinas, Roldan, & Beldi, 2021; WHO, 2021). However, there was inconsistency and a lack of specificity in recommendations about which organisational strategies would be most suitable within a hospital setting (Sanchez-Taltavull et al., 2021; WHO, 2021). We developed an alternate working model which proposed shift work and alternating teams for our department, but our proposal was rejected by management, and we were required to continue with a full staff complement on-site daily.

Promoting a safety culture through the development of organisational policies is important for contributing to a better mental health in the workplace (Devaraj, 2020; Hamouche, 2020; Yassi et al., 2007). Various protocols were introduced in our department to improve staff safety and ensure our adherence to infection control and safety compliance measures. Adherence was regularly monitored through audits by the STA Department’s infection control team, and continuous feedback was provided on improvement strategies. Signage was placed around our department to indicate the number of people per room to ensure adequate social distancing. Most of our meetings and activities were moved to a virtual platform in order to adhere to regulations and to keep staff members safe.

During the pandemic, a shortage of sanitisers, medical masks and toilet paper and the absence of guidelines on the use of PPE increased anxiety-related behaviours amongst healthcare professionals (Corkery & Maheshwari, 2020; Shigemura, Ursano, Morganstein, Kurosawa, & Benedek, 2020). Initially, PPE was provided according to our own risk assessment and availability of stock. At the early stages of the pandemic, there was a severe shortage of PPE (Le Roux & Dramowski, 2020; Zungu et al., 2021) and staff had to either purchase their own masks or wear the same mask for a few days. This was very distressing as we felt unsafe and uncared for within the health system. Once the hospital’s PPE committee was constituted and guidelines were developed according to the level of risk and exposure, the stress and anxiety experienced were reduced.

#### Staff well-being

Research has highlighted that the pandemic will have far-reaching psychological impacts including burnout, fatigue, moral injury, depression, increased anxiety and post-traumatic stress for healthcare professionals (Blake, Bermingham, Johnson, & Tabner, 2020; Robertson, Maposa, Somaroo, & Johnson, 2020; Sovold et al., 2021; Wong, Olusanya, Parulekar, & Highfield, 2021). Implementing and exploring the value of evidence-based self-care strategies empowers healthcare professionals to protect their well-being (Cocchiara et al., 2019; Sovold et al., 2021; Walton, Murray, & Christian, 2020). Staff activities focused on building resilience, offering support and implementing strategies to maintain mental well-being. Research has shown the importance of providing access to mental health support for healthcare professionals during a pandemic (Walton et al., 2020). Information was shared on free mental health support services, online applications and resources for healthcare professionals (e.g. Healthcare Workers Care Network and South African Depression and Anxiety Group and Care Pathways). Debriefing was organised for individuals and groups with the hospital’s Psychology Department. Support for COVID-positive staff members was provided through daily check-ins and sharing of resources on managing COVID-19 symptoms. An individualised response was developed for those who experienced loss. Discussions were held within the department on how to keep in touch with family and friends and how to minimise cross-infection to family members (Adams & Walls, 2020).

We created a space to share information on events such as online music festivals and ideas on self-care activities, movies, recipes and reading lists. Our well-being activities included mindfulness practice such as adult colouring, beadwork and gratitude practice by making gratitude jars, practising meditation and yoga. A graduation ceremony was hosted for our community service therapists who were denied this opportunity because of COVID-19. Research by Teo et al. (2021) found that teamwork and feeling appreciated at work were protective factors and assisted in mitigating the anxiety and stress associated with COVID-19.

Stigmatisation of healthcare professionals by their colleagues was rife during the early stages of COVID-19 (Grover, Singh, Sahoo, & Mehra, 2020). Regular and transparent communication about COVID-positive staff members, as well as patients, was important to decrease anxiety and fear associated with the unknown. Three of our staff members who tested positive in Wave 1 shared their story in a video produced by the Gauteng Department of Health. The aim of this video was to destigmatise being COVID-positive amongst healthcare professionals.

#### Interdependence of staff within a system

Interdependence within the clinical microsystem refers to the interaction of staff, characterised by collaboration, a willingness to help and a recognition that all contribute individually to a shared purpose (Johnson, 2010). As hospitals struggled with high patient loads during the pandemic, many healthcare professionals needed to act out of their scope of practice (Adams, Seedat, Coutts, & Kater, 2021; Bodiat & Rogers, 2020). We were called on to assist with swabbing patients and staff at designated points in the hospital. We assisted with packing PPE for staff and setting up a database for hospital staff COVID-19 infections. In addition, we were requested to accommodate the staff occupational health and safety service in our department. This consisted of telephonic risk assessments and follow-ups with positive staff members. The Head of Department was seconded onto the hospital’s vaccine committee to organise the vaccine roll-out for healthcare professionals. She also assisted with vaccine education and training sessions for staff and acted as a media liaison for the hospital. The STA Department assisted with queue management and registration for the vaccine roll-out. This intensive process took up our time, personnel and resources but was important within the timing of the overall development of the hospital’s response to the pandemic. Our collaborative approach assisted in relationship-building, networking and contributing to a historical event. An integrated approach to feeling valued needs to include training and development, maintaining staff safety and creating a psychologically safe space where every member feels a sense of belonging and purpose.

### Patients

**FIGURE 2:**
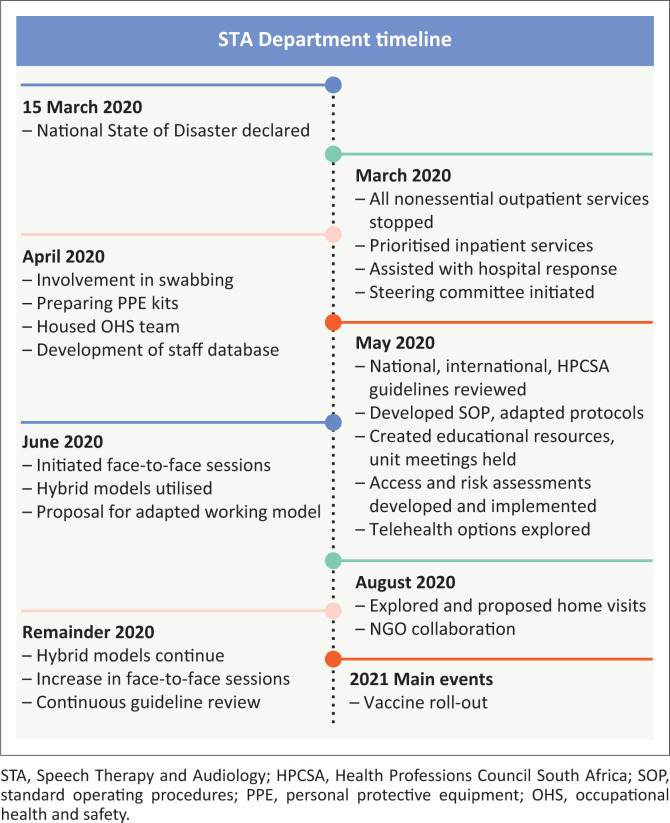
STA Department timeline.

Our services were aligned to the different levels of lockdown as indicated on the timeline in Figure 2. Rehabilitation is a human right (United Nations, 2006) and is identified as a key health strategy by the WHO (2019). The rehabilitation needs of persons with disabilities were not considered in many countries’ responses to the COVID-19 pandemic (Eskytė, Lawson, Orchad, & Andrews, 2020), as services were either stopped or curtailed (Negrini et al., 2020). Vulnerable people became even more vulnerable during this pandemic (Castelyn, Viljoen, Dhai, & Pepper, 2020). Our outpatient services were immediately stopped after the State of Disaster was declared in March 2020 (Dlamini Zuma, 2020; Moonasar et al., 2021). Our inpatient load was affected by the hospital’s decision to discharge all medically stable patients in order to free beds and increase staff availability for COVID-19 patients. This reprioritisation within the hospital for COVID-19 patients necessitated a meeting with medical heads of units to clarify referrals, discharge procedures and plan the clinical management of patients already in the hospital. Communication channels between teams were also discussed to improve the understanding of expectations and patient flow within and through the various units. Our protocols were adjusted to include prioritisation criteria and risk reduction strategies for the inpatient population. The criteria were based on guidelines developed by the Royal College of Speech and Language Therapists (2020) and recommendations made by West, Kevin and Welling (2020). Three levels of priority as indicated in Table 4 were developed and instituted to guide decision-making in Levels 5 and 4 of lockdown.

**TABLE 4:** Initial prioritisation guideline.

Level of priority	Speech therapy In and outpatients	Audiology In and outpatients
High priority	Swallowing assessment and management of severe oral and pharyngeal phase dysphagia Deep-partial, full-thickness and chemical facial burns at risk of contractures Laryngectomy patients if unable to be assisted telephonically (leaking speech valve, difficulty breathing or swallowing) Tracheostomised patients (blue dye assessments and speech valve trials were stopped)	Hearing assessment and management of patients with drug-resistant tuberculosis and meningitis Inpatients with severe-profound communication difficulty related to hearing loss Urgent repairs for all hearing devices Patients requiring emergency ENT surgical intervention Ototoxicity monitoring Cochlear implant workup and management of paediatrics, recently implanted patients and those with hearing loss secondary to meningitis
Medium priority	Communication disorders impacting patients’ abilities to indicate their needs in their immediate environment Oral dysphagia without risk of aspiration in newly diagnosed patients	Occupational audiology for patients at risk of losing employment Patients with chronic middle ear pathologies requiring baseline audiograms Baseline audiograms required for oncology initiation Neonatal hearing screening Workup of adult cochlear implant candidates and management of all cochlear implant recipients
Low priority	Oral dysphagia without risk of aspiration in previously diagnosed patients Patients who had at least one established mode of communication (e.g. AAC) Videofluoroscopy studies Patients who were able to implement home and ward exercise programmes independently or with caregiver assistance	Presbycutic hearing loss Patients previously fitted with a hearing device Annual cochlear implant follow-up

*Source*: Adapted from the Royal College of Speech & Language Therapists. (2020). *RCSLT guidance on Personal Protective Equipment (PPE) and COVID-19*; and West, J.S., Kevin, F.H., & Welling, B.D. (2020). Providing health care to patients with hearing loss during COVID-19 and physical distancing. *Laryngoscope Investigative Otolaryngology, 5*(3), 396–398. https://doi.org/10.1002/lio2.382

AAC, alternate and augmentative communication.

The above guidelines were continuously reviewed and adjusted according to emerging evidence and levels of lockdown. Risk reduction strategies included stopping high-risk procedures like speech-valve trials and modified Evans blue-dye tests (Cameron, Reynolds, & Zuidema, 1973 as cited in Swigert, 2003). The time spent with inpatients was reduced and the frequency of treatment was determined by the risk of transmission. All multidisciplinary joint treatment sessions were stopped to maintain social distancing. Other adjustments included the positioning of the therapist and ensuring that the appropriate PPE was used. Initially, the staff entering COVID-19 wards was restricted because of PPE and uncertainty about treatment protocols. Screening algorithms and augmentative and alternative communication boards were developed for doctors and nurses to facilitate communication with patients and safe eating and drinking practices. Note-writing templates were developed to reduce the time spent in wards. Research has shown that there was an increased need for information from family members of patients during this period as the levels of worry and uncertainty were high (Hugelius, Harada, & Marutani, 2021). Limited family contact because of visiting restrictions impacted family and caregiver training, education and counselling and obtaining relevant patient information. Despite the barriers discussed above, the STA Department continued clinical care and maintained a total of 17 310 contact sessions with speech and audiology inpatients.

Outpatient care was severely restricted because of lockdown regulations; however, our commitment to patient-centred care continued to motivate us to investigate other means of reaching our patients. The team regrouped to strategise approaches on how we could identify and meet the urgent needs of patients to maintain function in their home environments. We developed a proposal to obtain permission from the hospital to conduct home visits. However, because of the level of lockdown and concerns around staff safety, our request was denied. Missed opportunities to engage and partner with communities during COVID-19 were also reported by Rispel, Marshall, Matiwane and Tenza (2021). A non-governmental organisation was contacted to enquire if they were able to conduct the visits on our behalf. Care packages were developed for patients who required consumables and equipment to facilitate continued access. These included home programmes, batteries for patients with hearing aids, consumables for patients with head and neck cancer, Transpore tape and specialised feeding equipment for children with cleft lip and/or palate. Information for families and caregivers on how to stimulate communication at home, pamphlets on COVID-19 and mindfulness activities were included in the packs. The non-governmental organisation reported that they were well-received by patients who expressed their gratitude for the visit.

Numerous research studies have shown that people with disabilities have lower incomes and are at a heightened risk of food insecurity compared to people without disabilities (Banks et al., 2020; Park, Kim, Kim, Jeoung, & Park, 2020; The Lancet, 2020). Lockdown was especially difficult for already vulnerable groups (Abrahams, Boisits, Schneider, Prince, & Lund, 2021); some of the adult patients reported that they had limited access to food and others lived alone without familial support. The non-governmental organisation arranged food parcels for these patients and placed them on their database for follow-up visits. Feyisayo, Olufunke and Lambert (2021) found that an increasing number of South Africans became dependent on food parcels during the pandemic. Whilst the Gauteng Provincial Government identified social relief as a key response, it is evident that this may have been inaccessible to more vulnerable groups (Gauteng Provincial Government, 2020). The elderly and people with disabilities were expected to compete with others for limited resources within a context of restricted movement (De Groot & Lemanski, 2021).

Children’s exposure to formal and informal learning opportunities was limited because of lockdown restrictions (Murray, 2021). The disruption of rehabilitation services for children with disabilities negatively impacted their physical and mental well-being as well as their behavioural development (Allison & Levac, 2021; Rao, 2021). Because of these identified risks, we collaborated with Soweto TV to run a segment on how to stimulate language, literacy and play in the home during COVID-19. The segment was aired as part of the news bulletin of the day.

COVID-19 required us to investigate new and additional methods of service provision (Cacciante et al., 2021; Dixit et al., 2021). Our service package included face-to-face intervention, telehealth (asynchronous, synchronous and remote monitoring) or a hybrid model of incorporating the two. A study conducted by Camden and Silva (2021) across 76 countries showed that the use of telehealth by therapists had increased from 4% pre-COVID-19 to 70% by May 2020. The STA Department’s overall use of telehealth increased from 0% pre-COVID to 46% in 2021. Speech therapy for hearing-impaired children reached 88% during Level 5 lockdown and maintained 70% in 2021, with many patients electing to follow a hybrid model. A total of 520 synchronous and 632 asynchronous interventions were conducted from June 2020 until December 2021. An upward trend was noted in all other clinical areas in the STA Department.

Many studies have identified digital poverty as a barrier to the implementation of telehealth (Baker-Smith, Sood, Prospero, Zadokar, & Srivastava, 2016; Clare, 2021; The Lancet, 2020). Barriers include internal factors such as the lack of awareness and scepticism about change, technical readiness and external challenges such as costs, limited technical resources, data privacy issues and unclear liability of rehabilitation providers (Camden & Silva, 2021). Furthermore, Dixit et al. (2021) identified limited access to telehealth in patients with low income, limited English proficiency, low health, computer literacy and those accessing public healthcare. To understand our patients’ needs and preferences, we conducted a telehealth access questionnaire. This included questions related to data and Internet access, availability of smartphones, laptops and tablets as well as establishing if patients were interested in telehealth. These interviews highlighted the reality of digital poverty within our context, as most patients indicated their lack of data and internet. Whilst the identified barriers were overwhelming, we continued to look for solutions to overcome these. Internet access for both staff members and patients was the main challenge. We developed funding proposals to various private organisations but received no response. We also developed a comprehensive motivation to the hospital for improved Internet access as well as supporting equipment. Our proposal was rejected; however, this did not deter us. The high level of motivation and drive amongst staff members ensured that other creative solutions were developed; this included the use of personal resources and a mapping exercise to identify community centres and places that offered free Wi-Fi. We developed a roster for staff members to conduct telehealth sessions from home. Relevant accountability mechanisms were introduced to ensure transparency (e.g. the use of Google Calendars) and appropriate use of time. The Health Professionals Council of South Africa was contacted to discuss our plan to ensure compliance with the ethical rules and regulations. We developed telehealth record-keeping forms and created password-protected storage for videos sent in by patients for asynchronous sessions as well as recorded therapy sessions. Logistical issues such as patient payment for these sessions were discussed with our finance and patient affairs department.

A study conducted by Signal, Martin, Leys, Maloney and Bright (2020) found that most patients were unfamiliar with telehealth and sceptical of its value, but their attitudes changed once they received telehealth services. We are still refining our skills in providing synchronous telehealth sessions; however, many lessons have already been learnt, which have assisted in improving services. We have learnt that presession planning is important. This includes setting up the device, checking Internet stability, ensuring that all therapy resources are within the reach, communicating with patients and families on expectations for the session as well as what material would be required. Correct positioning and lighting are crucial and family routines and schedules must be accommodated. Patient and family feedback after each session is critical for continuity of care and achievement of goals. Effective implementation of telehealth requires organisational change and support (James et al., 2021). Sustainable telehealth implementation in the public health sector requires commitment of resources and logistical support from the Department of Health. We should continue to fine-tune, monitor and evaluate the implementation of telehealth (Abdel-Wahab, Rosenblatt, Prajogi, Zubizarretta, & Mikhail, 2020) in the public health sector.

Asynchronous intervention has an advantage over synchronous intervention by removing scheduling issues (Hill & Breslin, 2016). The efficiency of therapy can also be improved within a context of infrastructure limitations and language variability (Bhat, Kant, Vachhani, Rautara, & Kopparapu, 2020). Our asynchronous sessions required the development of digital learning resources, therapy programmes and educational material. We consulted an expert in digital health literacy who provided us with critical feedback on health literacy, high-impact messaging and technical aspects to improve the quality of our videos. These resources were shared through WhatsApp, email and short message service (SMS). Our overall goal of service delivery was to maintain patient-centred care in a safe environment for patients and therapists.

A strategic planning session with staff members identified the resources, processes and support that were required to provide services to patients who indicated a preference for face-to-face sessions. The adherence to non-pharmaceutical interventions to reduce the risk of infections was critical. These measures included patient screening, hand hygiene, booking fewer patients and increasing the time between patients to allow for cleaning. The number of people per room was limited and disinfection protocols were strictly followed (Agarwal et al., 2020; Cho et al., 2020). It was imperative that these infection control measures become a part of our routine clinical practice.

## Conclusion

At a macro level, the needs of people with disabilities should be visibly accounted for when planning a country’s response to a pandemic (De Biase et al., 2020). Leadership from the National and Provincial Rehabilitation Directorate needs to step in early to provide guidance to professionals to help facilitate their roles, responsibilities, priorities and functions in a pandemic to avoid the siloed response that was seen during the current pandemic (Maccarone & Masiero, 2021). Working in a pandemic requires adaptation, flexibility and innovation of healthcare models to enable service delivery (Pearlman, Tabaee, Sclafini, Sulica, & Selesnic, 2021). These advocated models need to be supported to guide implementation. Regular consultation and transparent and frequent communication with patients, families, healthcare professionals and communities are essential from the onset.

This article provided us with an opportunity to tell our story of adaptation, teamwork and resilience in a period of uncertainty. Storytelling allowed us to understand the meaning of our actions, our values and principles, ‘our fears and vulnerability, and the connective tissue that binds us together’ (Kumagia & Baruch, 2021, p. 1095). Our initial discomfort, confusion, fears and doubts about our role led us to redefine our purpose as healthcare professionals within an unprecedented and changing context. Research has found that healthcare professionals expressed a need to continue working and feeling a strong sense of purpose during the pandemic (Aughterson, McKinlay, Fancourt, & Burton, 2021). Our shared vision and collaborative decision-making provided a strong foundation in navigating the uncertainty. It was difficult when our ideas were not supported at higher levels, but we remained committed to finding innovative solutions within our context that aligned to our purpose and allowed us to continue patient care. According to Kumagia and Baruch (2021, p. 1095), stories ‘help us form empathic connections and focus on collective values and goals’. Networking and collaborating within our microsystem and mesosystem strengthened our response to staff and patient needs. The pandemic reminded us of the importance of compassionate leadership and responding to the socio-emotional needs within a team. Whilst we functioned as part of a broader, holistic system, it was important for us to remain ‘whole’ by focusing on our well-being.

Stories are being increasingly recognised for their potential as creators of change as they guide small, everyday decisions and practices (Essebo, 2021). We hope that our story will encourage others to tell theirs so that we could strengthen the narrative of practice within context. It was through this journey of storytelling that we gained a bird’s eye perspective of everything we had accomplished as a department in our response to COVID-19. This led us to submitting our application for the 7th Annual Batho Pele awards, where we were placed jointly in first position in the category of Best Responsive Government Institution. This recognition by the Ministry of the Department of Public Service and Administration has further increased the platforms on which we are able to continue telling our story.
